# Imaging Manifestations of a Subependymal Giant Cell Astrocytoma in Tuberous Sclerosis

**DOI:** 10.1155/2016/3750450

**Published:** 2016-01-31

**Authors:** Joseph R. Stein, Daniel A. Reidman

**Affiliations:** The Regional Medical Center, 3000 St. Matthews Road, Orangeburg, SC 29118, USA

## Abstract

Tuberous sclerosis is a rare genetic disorder resulting in benign tumor growth in various organs including the brain, heart, skin, eyes, kidney, and lung as well as systemic manifestations including seizures, cognitive impairment, and dermatologic abnormalities. This report shows the radiological findings and differentiation between a subependymal nodule and subependymal giant cell astrocytoma in a patient with tuberous sclerosis presenting with new onset seizures.

## 1. Introduction

Tuberous sclerosis complex (TSC) is an autosomal dominant genetic disorder resulting from mutations in the TSC1 gene or the TSC2 gene [1]. The disease is characterized by benign hamartoma development in several organs, including the brain, heart, skin, eyes, kidney, lung, and liver. The disease has an estimated prevalence between 1/6800 and 1/15000 [2–4]. Clinical manifestations of TSC seem to have an age-related expression pattern, as some manifestations are typically identifiable at birth while others develop later at different ages. Some patients may have a milder form of the disease and can remain undiagnosed while others can be debilitated by the disease from birth. Since the diagnosis of TS cannot be made based on a single clinical manifestation, careful attention to physical exam findings and radiologic abnormalities is vital for correct diagnosis. In this presentation, clinical and radiological findings of subependymal giant cell astrocytoma and cortical tubers leading to new onset seizures will be discussed.

## 2. Case Summary

A 26-year-old female with history of hypertension and dialysis-dependent ESRD with a GFR of 5 was admitted after having a seizure at work. Significant family history includes a father with tuberous sclerosis and brother with tuberous sclerosis and seizures. The patient stated she was at work the night before admission when she “felt hot” and started “shaking.” She described 1-2-second duration and serial, generalized myoclonic twitching lasting roughly 5 minutes prior to reported loss of consciousness. During EMS stabilization and transfer another generalized tonic-clonic convulsion occurred accompanied by postictal lethargy and confusion. The patient denied any prior history of seizures, cognitive impairment, or frequent headaches.

On examination vital signs revealed the patient to be tachycardic (164 beats per minute) and hypertensive (249 mmHg/153 mmHg). Neurological examination was normal. Several facial and body hyperpigmented fibrous plaques and a posterior lumbar Shagreen patch were noted.

## 3. Imaging Findings

A nonenhanced head computed tomography was performed to exclude ischemia or hemorrhage. This revealed a rounded, heterogeneous, soft tissue mass with central calcifications opposing the ependymal surface in the ventral body of the right lateral ventricle measuring approximately 12 mm × 18 m. No accompanying hydrocephalus or local neoplastic extension was evident.

Subsequent contrast enhanced brain MRI demonstrated a 23 mm × 23 mm × 18 mm round, well-circumscribed mass in the right lateral ventricle and roof of the third ventricle. The mass was heterogeneous with several internal signal voids. There was mild mass effect (4 mm septum pellucidum leftward shift), with adjacent white matter mild edema and gliosis. Scattered cortical based T2 and FLAIR hyperintensities were seen throughout both cerebral hemispheres, suggestive of cortical tubers.

Prior renal ultrasound and abdominal CT were obtained which demonstrated chronic cystic renal atrophy/chronic medical renal disease (Figures 4 and 5).

## 4. Discussion

The majority of TSC patients present with neurologic symptoms, with approximately 90% of affected individuals experiencing seizures and roughly half experiencing cognitive impairment, autism, or other behavioral disorders [5]. Epilepsy is seen in 70% to 90% of patients, most commonly presenting in the first year of life [6]. Autism spectrum disorder, intellectual disability, and other neurodevelopmental and psychiatric disorders associated with TSC also lead to a significant disability. Renal manifestations are the second most common finding associated with TS, with angiomyolipomas (AMLs) occurring in 80% and renal cystic disease in 50% of the patients [7]. Pulmonary involvement, specifically lymphangioleiomyomatosis (LAM), is the third most common cause of TSC associated morbidity, occurring in approximately 35% of reproductive age female TS patients [8, 9]. Cardiac rhabdomyoma can be found in 50%–65% of patients with TS [10]. Other features of TS include retinal nodular hamartomas, dental pits, gingival fibromas, rectal polyps, and bone cysts.

**Figure 1:**
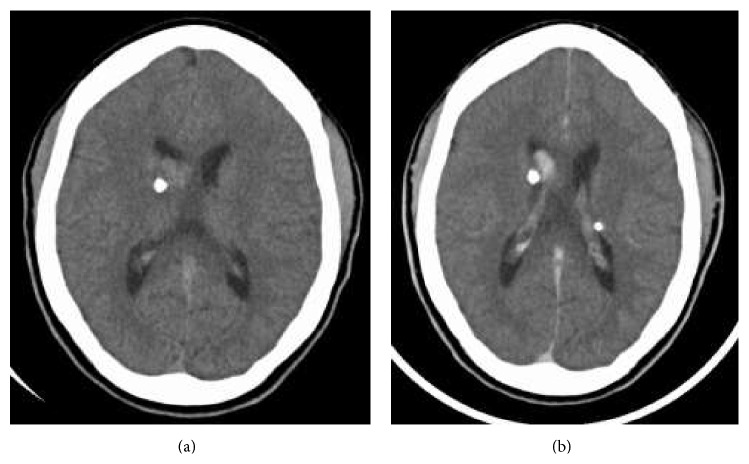
Axial noncontrast CT demonstrating a 12 × 18 mm partially calcified, enhancing soft tissue mass in the anterior body of the right lateral ventricle (a). Axial CT with contrast showing SEGA enhancement but no enhancement of tubers (b).

**Figure 2:**
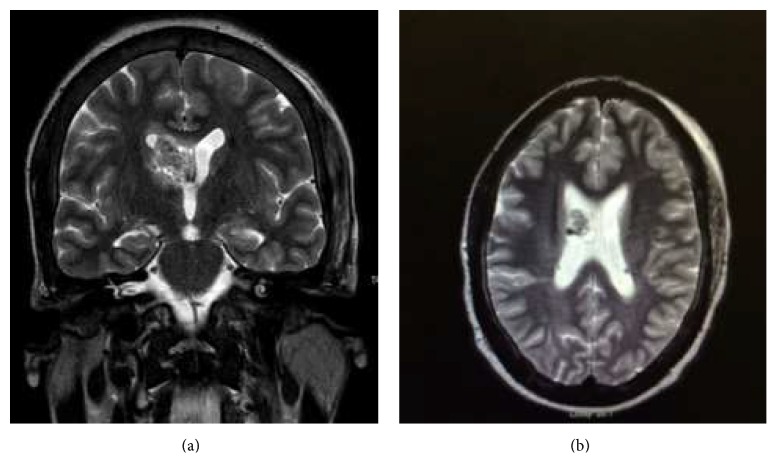
T2 weighted coronal (a) and axial (b) MRI showing 23 × 23 × 18 m lobulated mass in the right lateral ventricle and roof of the third ventricle which is isointense to grey matter and has small signal voids in keeping with calcifications.

**Figure 3:**
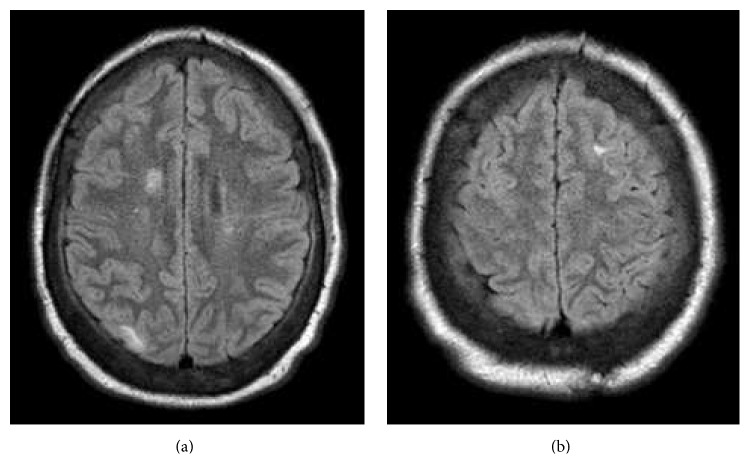
T2 FLAIR axial MRI showing subcortical areas of mixed signal mass in the right occipital cortex (a) and left frontal cortex (b), suggestive of cortical tubers.

**Figure 4:**
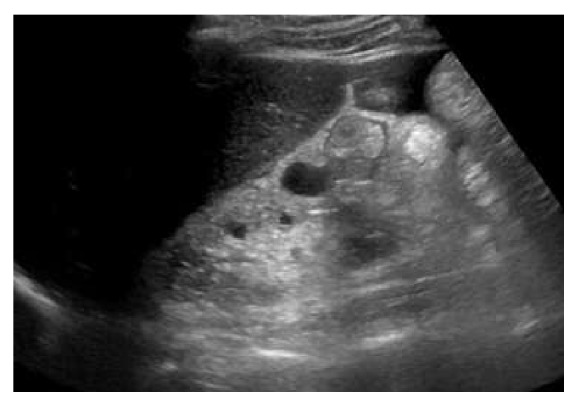
Renal US long R kidney demonstrating atrophy and cysts.

**Figure 5:**
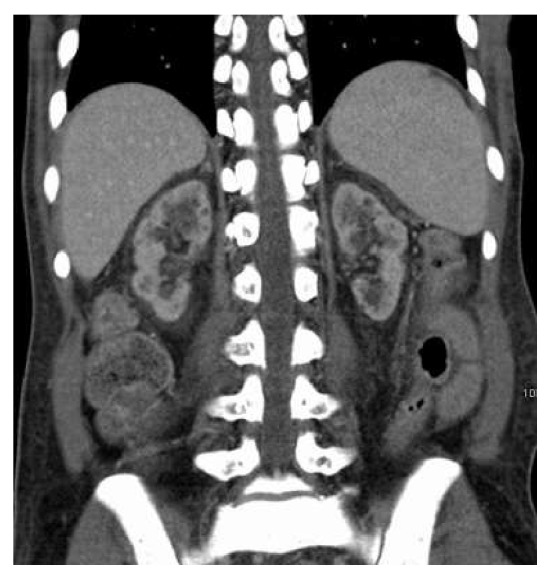
CT abdomen chronic cystic renal atrophy/chronic medical renal disease.

The main structural brain lesions include cortical tubers, subependymal nodules (SENs), and subependymal giant cell astrocytomas (SEGAs) [1, 5]. Cortical tubers develop prenatally and are seen in 90% of patients (Figure 1) [11]. Cortical tubers are a collection of giant cells, dysmorphic neurons, and gliosis that destroy the normal six-layer cortical structure [11]. There is a positive correlation between the number of cortical tubers and cognitive impairment and seizure control difficulty [12]. Magnetic resonance is the modality of choice for detecting cortical tubers. Increased signal intensity on T2-weighted images and decreased signal intensity on T1-weighted ones are demonstrated in almost all patients with the exception being infants and neonates where T1-weighted images show enhancement due to the similar relaxation time of T1 and the unmyelinated brain [13]. Only 10% of cortical tubers will be enhanced after administration of contrast material [13]. The patient in this case showed the typical findings of T2 and FLAIR hyperintensities in multiple areas suggesting cortical tubers, the likely etiology of her seizures (Figures 2 and 3).

Subependymal nodules are collections of abnormal, swollen glial cells and giant cells which cannot be differentiated as normal neural tissue. They have a tendency to calcify and can progress into subependymal giant cell astrocytomas, which are histologically indistinguishable from SENs but distinguishable based on their larger size, higher growth rate, and potential for mass effect compared to the relatively static course of SENs [14, 15]. The radiographic appearance of SENs on unenhanced CT will show small calcified foci along the wall of the lateral and third ventricle [13] (Figure 1). MR imaging will demonstrate hyperintensity on T1-weighted imaging and isointense or hyperintense T2 signal [13]. This variability is likely due to the extent of calcifications. SEGAs are slow growing tumors and most commonly manifest in the first two decades of life. Occurring in 1.7–26% of patients with TS, they consist of proliferative astrocytes and giant cells [11]. The most common location of SEGAs is the foramen of Monroe, leading to obstructive hydrocephalus and manifesting with signs and symptoms of increased intracranial pressure, but most are asymptomatic. Intermediate cells have been identified in SENs and SEGAs suggesting that SENs can transform into SEGAs [16]. Serial imaging can monitor growth of subependymal nodules and potential for transformation into SEGAs [16]. Compared to SENs, SEGAs demonstrate more intense enhancement on CT and are larger tumors (>1 cm) [17] (Figure 1). Suspicion for transformation should be considered if lesions measure 5 mm or greater in diameter, are incompletely calcified, and demonstrate enhancement [18]. There is also a reported increased frequency of malignant transformation in masses approximating the foramen of Monroe [18]. SEGAs show heterogenous enhancement on MR imaging, demonstrating T1 isointense and hypointense signal and T2/FLAIR isointense and hyperintense signal [19] (Figures 2 and 3). The imaging findings of this patient are suggestive of a subependymal giant cell astrocytoma versus a subependymal nodule based on its incomplete calcification, enhancement with gadolinium, and size (>1 cm). Surgical resection remains the recommended treatment for symptomatic SEGAs [20]. Surgery also remains the standard treatment for SEGAs demonstrating serial growth on neuroimaging, but experts indicate that some patients may benefit more from medical treatment depending on the size of the lesion [20]. Our patient's SEGA warranted neurosurgical consult given the above noted findings.

Generally speaking, tumors greater than 3 cm carry significant risk for complications and should be medically managed. Surgical intervention of SEGA > 3 cm carries a 67% risk of surgery-related complications and surgery on tumors > 4 cm was associated with a 73% risk of complications [21, 22]. Bilateral SEGAs, regardless their size, were associated with 67% risk of complications after surgery. No complications were observed in patients undergoing surgery for SEGAs < 2 cm. The 23 mm SEGA in our patient would likely benefit from surgical intervention and accordingly a neurosurgical consult was recommended. In the event in which surgical intervention is declined, serial monitoring of tumor growth in this patient would be based on clinical judgment. It is recommended that SEGAs are monitored serially with MRI every 1–3 years in patients younger than 25 as these tumors usually grow in children and adolescents but do not have a tendency to grow in adulthood [21, 22].

## 5. Conclusion

This case of a new onset seizure illustrated CNS manifestations of tuberous sclerosis. The patient presented with a new onset seizure and was found to have multiple cortical tubers and subependymal giant cell astrocytoma on brain imaging. The cortical tubers were the likely etiology of her seizure and the patient was placed on Keppra but more concerning was the astrocytoma mass in the body of the right lateral ventricle. The proximity to the right foramen of Monroe, its incomplete calcification, enhancement on MRI, and large size (>1 cm) make SEGA the likely diagnosis. A neurosurgery consult was recommended off-site, but surgery was not performed because of the patient's comorbidities and symptoms attributed to cortical tubers rather than symptomatic SEGA.
